# Influence of the equipment used for manual ventilation over the variability of respiratory mechanics in rabbits

**DOI:** 10.1186/cc10186

**Published:** 2011-06-22

**Authors:** CM Rebello, R Lorenzetti, LB Haddad, LA Vale, RS Mascaretti, R Quinzani, AD Deutsch

**Affiliations:** 1 Hospital Albert Einstein, São Paulo - SP, Brazil

## Introduction

A self-inflating bag is used for newborns' manual ventilation, using an oxygen concentration close to 100%, unknown peak inspiratory pressure (PIP), no end positive expiratory pressure (PEEP) and high tidal-volume (Vt). Manual ventilation using a T-piece device allows better control of PIP, use of PEEP and probably less variation in pulmonary ventilation.

## Objective

To compare using an experimental model with the adult rabbit, the variability of PIP, PEEP, Vt, minute ventilation (VMin), respiratory rate (RR), inspiratory time (Tins), expiratory time (Tex) and ratio Tins/Total time during manual ventilation using a self-inflating bag or T-piece device.

## Methods

Adult New Zealand White rabbits were manually ventilated by 21 individuals using a self-inflating bag (LIFESAVER^® ^Neonate Manual Resuscitator; Teleflex Medical, Research Triangle Park, NC, USA) or a T-piece device (Babypuff^®^; Fanem Ltd, São Paulo, Brazil). Before ventilation each animal was sedated with intramuscular ketamine-acepromazine solution (10 mg/kg and 0.1 mg/kg, respectively) and anesthetized (1% lidocaine, s.c.) at the site of incision for tracheostomy and carotid cannulation. After curarization (pancuronium 1 mg/kg, i.v.) the ventilation was started and ventilatory data (PIP, PEEP, Vt, minute volume, inspiratory and expiratory time) were continuously recorded until sacrifice with sodium pentobarbital (100 mg/kg, i.v.), after 10-minute ventilation. For each variable analyzed a variability index was calculated, defined as the standard deviation of the mean values of each variable during the 10-minute ventilation. Statistical analysis was by *t *test or Mann-Whitney test, significance was set at *P *= 0.05.

## Results

The variability indices for all variables analyzed during the 10-minute ventilation are shown in Table 1.

**Table 1:** 

	T-piece (*n *= 21)	Self-inflating-bag (*n *= 21)	*P *value
Vt (ml/kg)	0.7 ± 0.7	2.5 ± 1.6	<0.001
VMin (ml/kg)	28.7 ± 14.6	122.3 ± 86.2	<0.001
PIP (cmH_2_O)	0.34 ± 0.21	3.3 ± 2.1	<0.001
PEEP (cmH_2_O)	0.2 ± 0.2	0.0 ± 0.0	<0.001
RR (bpm)	2.6 ± 1.3	9.9 ± 25.6	0.087
Tins (seg)	0.12 ± 0.05	0.13 ± 0.23	<0.001
Tex (seg)	0.13 ± 0.06	0.20 ± 0.22	0.902
Tins/Tt	0.04 ± 0.01	0.06 ± 0.07	0.034

Data are mean ± SD.

## Conclusion

The authors conclude that the use of a T-piece device allows lower variability during manual ventilation, with the exception of respiratory rate and expiratory time. We speculate that this lower variability could result in lower lung injury during manual ventilation.

